# The effects of sex hormones on BDNF secretion in airway smooth muscle of patients with obstructive pulmonary disease

**DOI:** 10.1016/j.heliyon.2025.e42688

**Published:** 2025-02-13

**Authors:** Shengyu Wang, Zihe Cheng, Siyu Li, Yao Tian, Jing Zhou, Min Yang

**Affiliations:** Department of Pulmonary and Critical Care Medicine, The First Affiliated Hospital of Xi'an Medical University, Xi’an Medical University, Xi’an, China

**Keywords:** BDNF, Hormonal regulation, Airway smooth muscle, Obstructive pulmonary disease

## Abstract

Obstructive pulmonary diseases, including asthma and chronic obstructive pulmonary disease, present significant global public health challenges and substantially impair patients' quality of life. Research indicates that sex hormones and brain-derived neurotrophic factor (BDNF) are pivotal in regulating airway function and inflammatory responses. Specifically, sex hormones modulate the expression and function of BDNF through their receptors, namely estrogen receptors and androgen receptors. Conversely, BDNF enhances cell survival and exerts anti-inflammatory and antioxidant effects, influencing the functionality of sex hormone receptors. This review elucidates the interactions between sex hormones and BDNF in obstructive pulmonary disease, emphasizing their synergistic roles in managing airway inflammation, facilitating tissue repair, and mitigating oxidative stress. Through a thorough examination of these mechanisms, this review aims to foster a more profound comprehension of the potential therapeutic implications for the management of these diseases.

## 1 Introduction

Obstructive pulmonary diseases, including asthma and chronic obstructive pulmonary disease (COPD), represent significant global public health challenges. According to the World Health Organization (WHO), COPD is the third leading cause of death globally, affecting over 250 million individuals, while asthma impacts more than 300 million people [1,2]. These conditions are characterized by airway obstruction, airflow limitation, chronic inflammation, and structural remodeling, all of which considerably diminish patients' quality of life and impose a substantial burden on healthcare systems worldwide [3]. Despite the availability of various treatment modalities, the pathophysiological mechanisms underlying obstructive pulmonary diseases remain inadequately understood, highlighting the urgent need for further research to develop more effective therapeutic strategies.

### 1.1 The potential role of BDNF in airway smooth muscle

Brain-derived neurotrophic factor (BDNF) is a crucial neurotrophic factor initially identified in the central nervous system, where it primarily sustains to neuronal survival, differentiation, and synaptic plasticity [4]. Recent evidence has increasingly demonstrated the expression of BDNF and its receptors in peripheral tissues, including airway smooth muscle within the lungs [5]. Although the mechanisms underlying BDNF's role in airway smooth muscle are not fully understood, studies indicate that it is instrumental in regulating airway reactivity and structural remodeling by modulating calcium ion influx and influencing the contraction and proliferation of smooth muscle cells [6,7]. Consequently, further exploration of BDNF's functions and regulatory mechanisms in airway smooth muscle may enhance our understanding of the pathogenesis of obstructive pulmonary diseases and offer new avenues for therapeutic intervention.

### 1.2 The importance of sex hormones in regulating BDNF

Sex hormones, including estrogens and androgens, play key roles in various physiological processes. The presence of sex hormone receptors in lung tissues indicates the potential for these hormones to regulate local physiological functions [8]. Research indicated that sex hormones can modulate the expression and function of BDNF through multiple pathways. For instance, estrogen can enhance BDNF gene transcription and protein synthesis by activating estrogen receptors [9]. Additionally, sex hormones may indirectly affect BDNF activity by modulating inflammatory mediators, oxidative stress, and cellular signaling pathways [10]. Understanding the regulatory mechanisms by which sex hormones affect BDNF is essential for elucidating their role in obstructive pulmonary diseases.

This review aims to review the effects of sex hormones on BDNF secretion in the airway smooth muscle of patients with obstructive pulmonary disease. Through an analysis of existing literature, we will explore the mechanisms by which BDNF operates in airway smooth muscle, examine how sex hormones regulate BDNF expression and function, and discuss the potential therapeutic implications of sex hormones in obstructive pulmonary diseases.

## 2 Biological functions of BDNF

### 2.1 Structure, function, and signal transduction mechanisms of BDNF

BDNF is a multifunctional protein abundantly expressed in both the central nervous system (CNS) and peripheral tissues, classified within the neurotrophic factor family. The initial synthesis of BDNF occurs in an inactive precursor form, known as pro-BDNF. Pro-BDNF is a larger, inactive molecule that requires processing to become biologically active. This processing occurs through the action of proteolytic enzymes, which cleave pro-BDNF to produce the mature form of the protein. The mature BDNF consists of 119 amino acids and retains a highly conserved structure that is critical for its function [11].

Once converted into its mature form, BDNF exerts its biological effects by binding to its high-affinity receptor, Tropomyosin receptor kinase B (TrkB), which is predominantly expressed in neurons but can also be found in other cell types. TrkB, a transmembrane tyrosine kinase receptor, is predominantly expressed in neurons but is also present in various other cell types. Upon binding to BDNF, TrkB undergoes autophosphorylation of its tyrosine residues, which subsequently activating multiple signaling pathways, including the PI3K/Akt pathway, the MAPK/ERK pathway, and the PLC-γ pathway [12].

PI3K/Akt Pathway: This pathway is primarily involved in promoting cell survival and inhibiting apoptotic processes. Activation of PI3K leads to the production of PIP3, which subsequently activates Akt. Activated Akt then phosphorylates and inhibits several pro-apoptotic factors, including BAD and Caspase-9, thereby promoting cell survival [13].

MAPK/ERK Pathway: This pathway mainly regulates cell proliferation and differentiation. The phosphorylation of the TrkB receptor activates the Ras protein, which sequentially activates Raf, MEK, and ERK. Once activated, ERK translocates into the nucleus, where it phosphorylates various transcription factors, ultimately regulating gene expression to promote cell proliferation and differentiation [4].

PLC-γ Pathway: This pathway plays a critical role in the regulation of intracellular calcium ion concentration. The activation of PLC-γ hydrolyzes PIP2 to produce inositol trisphosphate (IP3) and diacylglycerol (DAG). IP3 induces the release of calcium ions from the endoplasmic reticulum, while DAG activates protein kinase C (PKC), together orchestrating various cellular functions [5].

Additionally, BDNF can interact with the low-affinity receptor p75NTR, a member of the tumor necrosis factor receptor superfamily. Although p75NTR lacks intrinsic enzymatic activity, it has the capacity to recruit signaling adaptor proteins that modulate TrkB signaling. Furthermore, it may induce apoptosis by activating pathways such as JNK and RhoA [14].

### 2.2 Roles of BDNF in the nervous system and other tissues

BDNF plays a pivotal role in a multitude of processes within the CNS. It is crucial for neuronal development and maturation, as well as for maintaining synaptic plasticity and neuronal survival in the adult nervous system. BDNF enhances synaptic transmission, promotes long-term potentiation (LTP), and stabilizes postsynaptic structures, all of which contribute to learning and memory processes. Additionally, BDNF is recognized for its neuroprotective effects in response to stress and in neurodegenerative diseases, such as Alzheimer's disease and Parkinson's disease [15].

In peripheral tissues, BDNF's functions are equally important. It is expressed in various tissues, including muscle, heart, liver, and lungs, where it regulates numerous physiological processes. For instance, in muscle tissue, BDNF promotes muscle cell growth and repair; while in the heart, it supports the survival and function of cardiomyocytes [16]. In the lungs, BDNF is involved in the regulation of airway smooth muscle, potentially influencing airway contraction and remodeling by modulating calcium ion influx and cell proliferation [6].

In conclusion, BDNF interacts with its receptors, TrkB and p75NTR, to initiate a series of complex signaling pathways that regulate diverse functions in the nervous system and peripheral tissues. A deeper comprehension of the biological functions and signaling mechanisms of BDNF not only enriches our understanding of its roles in health and disease but also offers invaluable insights into the development of novel therapeutic strategies.

## 3 Expression of BDNF in airway smooth muscle

### 3.1 Expression and regulation of BDNF in airway smooth muscle

BDNF was initially identified in the central nervous system; however, emerging research has demonstrated that BDNF and its receptors are also widely expressed in peripheral tissues, particularly in airway smooth muscle (ASM). ASM cells are essential components of airway structure and function, and they play significant roles in respiratory diseases.

Expression: The expression of BDNF in ASM is primarily assessed through immunohistochemistry and Western blot techniques. Studies indicate that BDNF is expressed in ASM cells across both humans and animal models [6,7]. It is noteworthy that BDNF expression is significantly elevated in the ASM of patients with asthma, suggesting its potential role in airway remodeling and hyperreactivity [17].

Regulation: The expression of BDNF is subject to modulation by various factors, including inflammatory cytokines, oxidative stress, and neuropeptides. For instance, inflammatory conditions can significantly increase BDNF expression in ASM cells by enhancing specific signaling pathways including plasma membrane Ca2+ regulatory mechanisms [18]. Moreover, oxidative stress, particularly that resulting from cigarette smoke exposure, has been demonstrated to enhance BDNF secretion by regulating TRPC channels [19]. Neuropeptides, such as substance P, have also been shown to upregulate BDNF expression, underscoring the complex interplay between the nervous system and BDNF in ASM [20].

### 3.2 Role of BDNF in airway smooth muscle function and pathological processes

BDNF functions in ASM primarily through its receptors, TrkB and p75NTR.

#### 3.2.1 Regulation of airway smooth muscle contraction

BDNF binds to the TrkB receptor, activating downstream pathways, including the PLC-γ and MAPK/ERK pathways. These pathways are critical in regulating intracellular calcium ion concentration in ASM cells, thereby influencing muscle contraction [21]. Research has demonstrated that exogenous BDNF enhances the responsiveness of ASM to contractile agonists like acetylcholine, increasing airway resistance [22]. This effect is particularly pronounced in asthma patients, suggesting that BDNF may play a significant role in airway hyperreactivity.

#### 3.2.2 Promotion of airway smooth muscle cell proliferation and migration

In addition to its function in muscle contraction, BDNF has been demonstrated to facilitate the proliferation and migration of ASM cells through the activation of the PI3K/Akt and MAPK/ERK pathways [23]. This function is especially critical in the context of airway remodeling, a hallmark of diseases like asthma and COPD, which is characterized by the thickening of the ASM layer and abnormal deposition of the extracellular matrix [24]. The promotion of smooth muscle cell proliferation and migration by BDNF accelerates airway remodeling, thereby exacerbating airway obstruction and contributing to breathing difficulties.

#### 3.2.3 Regulation of inflammatory responses and apoptosis

BDNF plays a crucial role in regulating inflammatory responses and apoptosis in ASM. Research indicates that BDNF can induce apoptosis by activating the JNK and RhoA pathways through the p75NTR receptor [14]. Additionally, BDNF modulates the expression of inflammatory cytokines in ASM cells, contributing to airway inflammatory responses [25]. This involvement is s of particular significance in the context of airway inflammation and remodeling.

The expression and regulation of BDNF in ASM, along with its roles in ASM function and pathological processes, highlight the potential importance of BDNF in respiratory diseases. A comprehensive investigation into the mechanisms of BDNF may facilitate the development of novel therapeutic strategies aimed at improving the prognosis of patients with asthma and COPD.

## 4 Regulatory mechanisms of sex hormones on BDNF secretion

### 4.1 Regulation of BDNF by estrogen

**Fig. 1:**
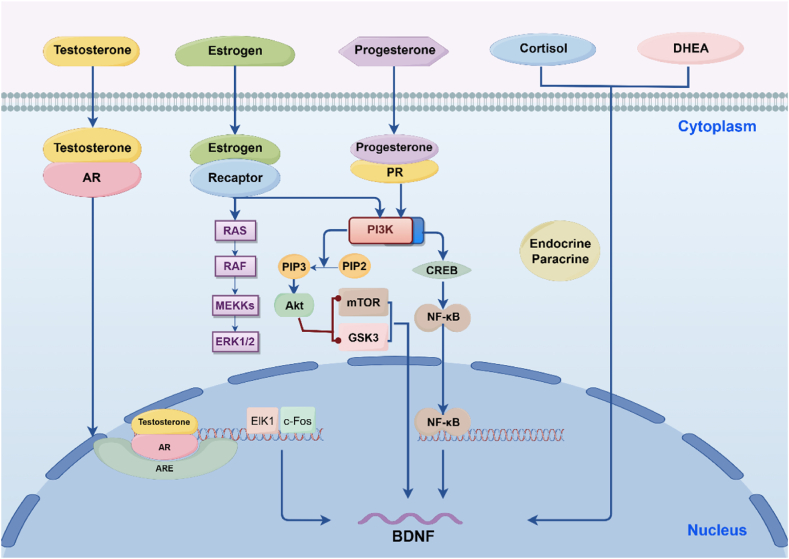
Mechanisms of sex hormones influence on BDNF secretion.

Estrogen, as an important sex hormone, regulates the expression of BDNF through its receptors ERα and ERβ, initiating a series of intracellular signaling pathways (Fig. 1).

#### 4.1.1 ERK/MAPK signaling pathway

ERα/ERβ Receptor Binding: Estrogen binds to ERα or ERβ receptors, triggering the activation of the ERK/MAPK signaling pathway. ERα and ERβ bind to estrogen response elements (EREs) in the promoter regions of genes in the nucleus, initiating the expression of transcription factors such as Elk-1 and c-Fos [26].

ERK/MAPK Activation: The receptor-ligand complex activates the Ras-Raf-MEK-ERK signaling cascade, leading to the phosphorylation and activation of ERK1/2. Activated ERK1/2 enters the nucleus, promoting the phosphorylation and activation of transcription factors [27].

Transcriptional Regulation: Activated ERK1/2 enhances the expression of transcription factors like Elk-1 and c-Fos, which subsequently bind to the promoter region of the BDNF gene, increasing BDNF gene transcription and thus upregulating BDNF expression. Notably, studies on Tenascin-C have demonstrated that ERK1/2 activation can phosphorylate and activate Elk-1 and c-Fos, promoting the expression of genes related to cell proliferation [28].

#### 4.1.2 PI3K/Akt signaling pathway

Estrogen also activates the PI3K/Akt signaling pathway by binding to ERα or ERβ receptors. This process involves the binding of estrogen receptors (ER) to the p85 regulatory subunit of PI3K, leading to the recruitment and activation of PI3K. Consequently, PI3K phosphorylates phosphatidylinositol bisphosphate (PIP2) to phosphatidylinositol trisphosphate (PIP3), further activating downstream Akt. Activated Akt phosphorylates various downstream effectors, such as mTOR and GSK-3β, ultimately promoting the expression and secretion of BDNF [29,30].

### 4.2 Regulation of BDNF by testosterone

Testosterone has been suggested to regulate BDNF expression in various tissues and under different pathological conditions. This regulatory process is hypothesized to occur primarily through the interaction between testosterone and androgen receptors (AR), which may influence BDNF gene expression and secretion.

#### 4.2.1 Role in Normal tissues

AR Receptor Binding: Testosterone binds to intracellular AR, forming a receptor complex that translocases to the nucleus [31].

AR-DNA Binding: The receptor complex binds to androgen response elements (AREs) in the nucleus, activating the transcription of the BDNF gene [32]. This process involves the binding of AR to specific sequences on DNA, thereby facilitating the regulation of gene expression.

Transcriptional Regulation: The activation of the AR complex culminates in the enhanced transcription of BDNF mRNA, promoting the synthesis and secretion of BDNF protein. This regulatory mechanism is observable in both neural and non-neural tissues, underpinning the diverse physiological roles of testosterone [32].

### 4.3 Regulation of BDNF by other sex hormones

In addition to estrogen and testosterone, it is crucial to acknowledge that other sex hormones also play a significant role in regulating BDNF secretion.

#### 4.3.1 Regulation by progesterone

Receptor Binding and Signaling: Progesterone exerts its biological effects primarily through binding to progesterone receptors (PR), which triggers a series of intracellular signaling cascades, notably including the MAPK and PI3K/Akt pathways. This interaction is crucial in modulating BDNF expression [33].

Transcription Factor Regulation: Upon activation, progesterone receptors affect the activity of various transcription factors, including CREB and NF-κB, which bind to the promoter region of the BDNF gene and regulate its transcription [34].

Neuroprotective Effects: Progesterone has been shown to upregulate BDNF expression, contributing to neuroprotective mechanisms that foster neuronal survival and enhance synaptic plasticity. These effects are observed in both the central and peripheral nervous systems [33].

#### 4.3.2 Effects of other sex hormones

Steroid hormones such as cortisol and dehydroepiandrosterone (DHEA) also regulate BDNF expression through complex endocrine and paracrine mechanisms. For example, the synergistic action of estrogen and progesterone may influence BDNF expression under certain physiological and pathological conditions [34].

These insights provide essential theoretical underpinnings that could pave the way for the development of novel therapeutic strategies aimed at addressing neurodegenerative diseases and respiratory conditions linked to BDNF dysregulation.

### 4.4 The role of sex hormones in obstructive pulmonary diseases

#### 4.4.1 Mechanisms of sex hormones in asthma and COPD

Obstructive pulmonary diseases, such as asthma and COPD, are chronic respiratory conditions characterized by airway obstruction and airflow limitation. The mechanisms by which sex hormones influence these diseases are complex, involving multiple cellular and molecular pathways. The modulation of inflammation, airway remodeling, oxidative stress and tissue regeneration by sex hormones is critical for understanding disease progression and treatment outcomes in both asthma and COPD patients. Here we briefly review the underlying mechanisms of how sex hormones (estrogens and androgens) influence asthma and COPD.

#### 4.4.2 Role of estrogen in asthma and COPD

Inflammation Regulation: Estrogen exerts complex effects on inflammation, which can vary depending on the tissue, cell type, and the specific cytokines involved. In immune cells such as macrophages, eosinophils, and lymphocytes, estrogen primarily exerts anti-inflammatory effects through its receptors ERα and ERβ. Studies have shown that the activation of ERα typically results in the downregulation of pro-inflammatory cytokines, such as TNF-α and IL-6, potentially reducing airway inflammation and hyperreactivity. In contrast, the activation of ERβ may have a more pronounced effect in inhibiting these pro-inflammatory cytokines [35]. These receptor-specific effects suggest that the overall anti-inflammatory role of estrogen in asthma may be influenced by the balance between ERα and ERβ activation. However, in certain contexts, particularly at high concentrations or in seosinophilia, estrogen may stimulate the production of pro-inflammatory cytokines, such as IL-5 [36]. The role of estrogen in airway inflammation is also modulated by factors such as sex, with differences observed between males and females, particularly during different stages of the menstrual cycle and hormonal fluctuations. Further investigation is needed to fully elucidate the nuanced role of estrogen and its receptors in asthma pathogenesis.

Airway Remodeling: Estrogen plays a significant role in airway remodeling, a process characterized by airway smooth muscle (ASM) cell proliferation, fibroblast activation, and extracellular matrix (ECM) deposition. Studies suggest that estrogen may inhibit ASM cell proliferation, potentially slowing airway remodeling [37]. Interestingly, the effects of estrogen on airway remodeling are receptor-dependent. Activation of ERα has been associated with the suppression of ASM cell proliferation, whereas ERβ activation may play a more prominent role in regulating fibroblast activation and ECM deposition [38]. This activity mitigates abnormal airway structural changes and reduces airway remodeling.

Oxidative Stress: Estrogen enhances the expression and activity of antioxidant enzymes, which is vital for reducing oxidative stress-induced airway damage. This protective role is essential for alleviating asthma symptoms and preventing disease exacerbation. Research indicates that estrogen can increase the activity of superoxide dismutase (SOD) and glutathione peroxidase (GPx), lowering oxidative stress levels and protecting airway cells [39].

#### 4.4.3 Role of androgens in asthma and COPD

Anti-inflammatory Effects: Androgens exert anti-inflammatory effects on immune cells through AR, regulating inflammatory responses. Androgens can inhibit the activity of the NF-κB signaling pathway, reducing the release of pro-inflammatory cytokines such as IL-8 and MCP-1, thus alleviating airway inflammation in COPD patients [40]. Androgens also reduce inflammatory responses and cell apoptosis by modulating MAPK and PI3K/Akt pathways [41].

Lung Function Protection: Androgens enhance the repair capacity of bronchial epithelial cells and promote lung tissue regeneration. Studies have shown that androgen therapy can improve lung function and reduce airway obstruction in COPD patients [34]. Furthermore, androgens promote the proliferation and differentiation of bronchial epithelial cells through AR-mediated signaling pathways, enhancing their barrier function [33]. This regenerative capacity of androgens may explain the observed improvement in COPD symptoms and lung function in patients undergoing androgen replacement therapy.

Muscle Protection: COPD patients often suffer from muscle wasting and weakness, which adversely affects their quality of life. Androgens can increase muscle mass and strength by promoting protein synthesis, which improves the overall health of COPD patients [42,43]. This is achieved through the activation of the mTOR signaling pathway, a critical regulator of muscle protein synthesis and growth [33].

## 5 Conclusion

This review explores the complex relationships between sex hormones, BDNF, and obstructive pulmonary diseases. Sex hormones regulate the expression and function of BDNF through their receptors (ER and AR), while BDNF, by promoting cell survival, anti-inflammatory, and antioxidant effects, feedback regulates the function of sex hormone receptors. The synergistic interaction between sex hormones and BDNF plays a crucial role in the modulation of airway inflammation, tissue repair processes, and oxidative stress, ultimately impacting the progression and symptomatic manifestations of obstructive pulmonary diseases. While BDNF has neuroprotective effects in conditions like Alzheimer's and Parkinson's disease, its role in airway diseases is multifaceted and context-dependent. Although current research highlights the significant roles of sex hormones and BDNF in airway diseases, several limitations remain. These include insufficient mechanistic studies, lack of robust clinical data, and small sample sizes in existing trials. In conclusion, while BDNF presents a promising therapeutic target in airway diseases, its role remains complex and requires further investigation. A detailed exploration of the mechanisms by which BDNF influences airway inflammation, remodeling, and tissue repair, combined with large-scale clinical trials and the development of novel treatment strategies, will be critical in advancing our understanding and treatment of obstructive pulmonary diseases. Such advancements can significantly enhance patient outcomes and improve overall quality of life.

## CRediT authorship contribution statement

**Shengyu Wang:** Writing – original draft, Resources, Conceptualization. **Zihe Cheng:** Writing – review & editing. **Siyu Li:** Writing – review & editing. **Yao Tian:** Investigation. **Jing Zhou:** Supervision. **Min Yang:** Conceptualization.

## Informed consent statement

Not applicable.

## Institutional review board statement

Not applicable.

## Data availability statement

Data sharing is not applicable to this article as no new data were created or analyzed in this study.

## Ethics statement

The authors declare that this manuscript does not involve any ethical issues related to human subjects, animals, or conflicts of interest. No experiments or studies involving humans or animals were conducted for this research.

**Table:** Abbreviations

AR	androgen receptor
ARE	androgen response element
ASM	airway smooth muscle
BDNF	brain-derived neurotrophic factor
CNS	central nervous system
COPD	chronic obstructive pulmonary disease
DAG	diacylglycerol
DHEA	dehydroepiandrosterone
ER	estrogen receptor
ERE	estrogen response element
GPx	glutathione peroxidase
IP3	inositol trisphosphate
LTP	long-term potentiation
PIP2	phosphatidylinositol bisphosphate
PIP3	phosphatidylinositol trisphosphate
PKC	protein kinase C
PR	progesterone receptor
SOD	superoxide dismutase
TrkB	Tropomyosin receptor kinase B
WHO	World Health Organization

## Funding

Foundation and Transformation of Prevention and Treatment of Acute Lung Injury (Shaanxi Jiaohan [2024] No. 21); Acute Lung Injury Prevention and Treatment Foundation and Transformation Innovation Team (Shaanxi Jiaohan [2023] No. 997); Shaanxi Province Key Program Fund (2017SF-256).

## Declaration of competing interest

The authors declare that they have no known competing financial interests or personal relationships that could have appeared to influence the work reported in this paper.
